# Eccrine porocarcinoma: an extremely rare cutaneous tumor from a radiological point of view – case report and review of the literature

**DOI:** 10.1259/bjrcr.20220044

**Published:** 2022-11-01

**Authors:** Imane Mahdar, Ghizlane Lembarki, Jihad Jamil, Hiba Safi-eddine, Belmoudden Najib, Mouna Sabiri, Mohamed Labied, Samia El Manjra, Lezar Samira, Mounia Diouri, Fatiha Essodegui

**Affiliations:** 1 Central Unit of Radiology, Ibn Rochd University Hospital, Casablanca, Morocco; 2 Plastic Surgery Center, Ibn Rochd University Hospital, Casablanca, Morocco

## Abstract

Eccrine porocarcinoma is a rare type of skin neoplasm. It represents less than 0.01% of all epithelial cutaneous tumors. Early diagnosis is the only way to minimize the mortality rate, given its aggressive nature and the high rate of local recurrence and metastasis. Clinical diagnosis is challenging and the confirmation is histological. Few studies have been published about the radiological features of eccrine porocarcinoma. We report a case of a localized eccrine porocarcinoma along with ultrasound, MRI features, and a review of the literature to highlight the role of imaging in the diagnosis and treatment plan.

## Introduction

Eccrine porocarcinoma (EPC) is a rare type of malignant cutaneous neoplasm. The clinical presentation is non-specific which can make the diagnosis quite challenging, as it is usually mistaken for other skin neoplasms. A biopsy is required for the confirmation, even though imaging modalities can help guide the diagnosis and provide valuable information about the extension. Surgical excision with large margins is the treatment of choice in local presentations, whereas metastatic cases are treated by chemotherapy.

## Case report

### Clinical presentation

**Figure 1.**
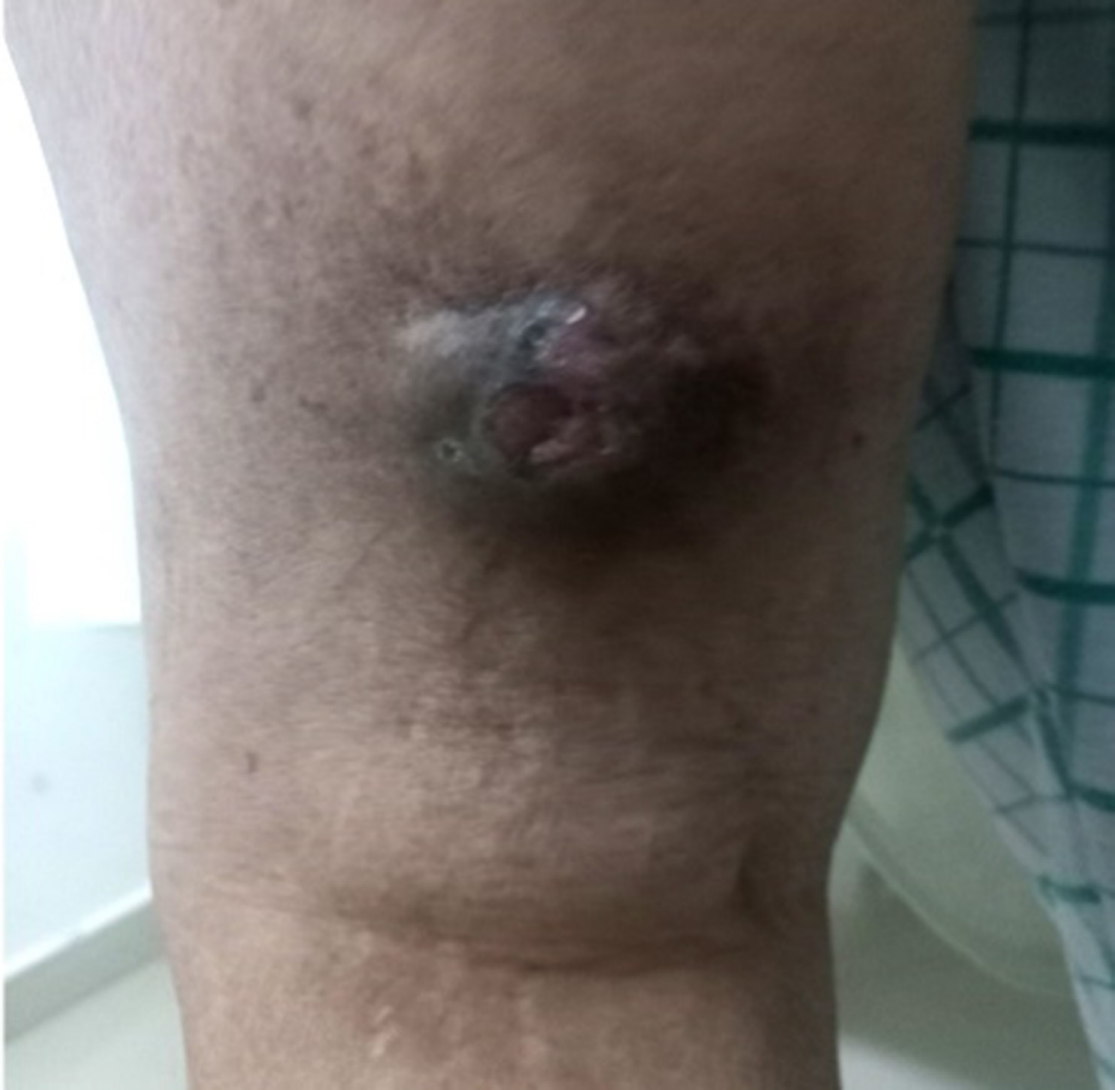
Clinical photography of the lesion on the left thigh, showing a well-defined pigmented mass with central ulcerations.

We present the case of an 87-year-old female with no prior medical history, presented with a painless mass on the left thigh, that has been progressing for over 10 years. It significantly increased in size in the last 3 months and became painful. Clinical examination revealed a 5 × 5.5 cm rounded, pigmented, well-demarcated, hard lesion, with central ulcerations and focal hemorrhages, located in the inferior region of the posterior aspect of the left thigh with restricted mobility (Figure 1). No other lesions were found elsewhere and no lymph nodes were clinically involved. Fever, anorexia, and weight loss were not reported. The patient consulted a dermatologist. On blood tests, no significant abnormality was found.

### Differential diagnosis

Porocarcinoma presents as an erythematous or violaceous mass or nodule, it can be misinterpreted as another type of skin neoplasm particularly squamous or basal cell carcinomas which can have a similar clinical presentation or even some benign skin tumors. Other differential diagnosis included cutaneous B-cell lymphoma, nodular melanoma and other adnexal cutaneous tumors.

### Investigations

**Figure 2.**
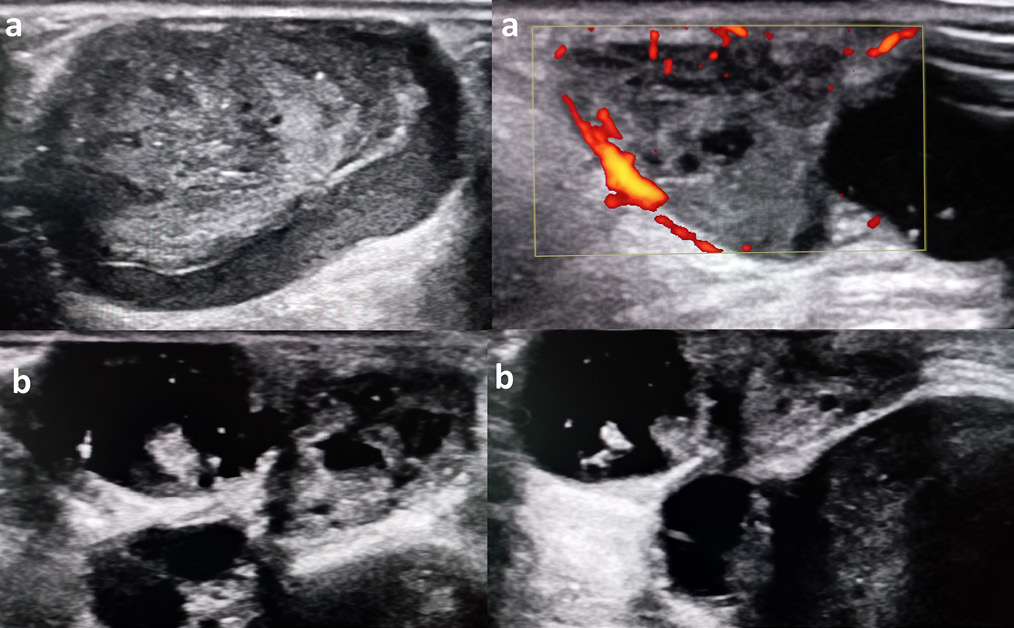
Ultrasound images showing a multilobulated oval-shaped mass of the cutaneous and subcutaneous tissue, with heterogeneous solid components (**a**) and cystic components, some of these cysts contain internal echoes (**b**).

**Figure 3.**
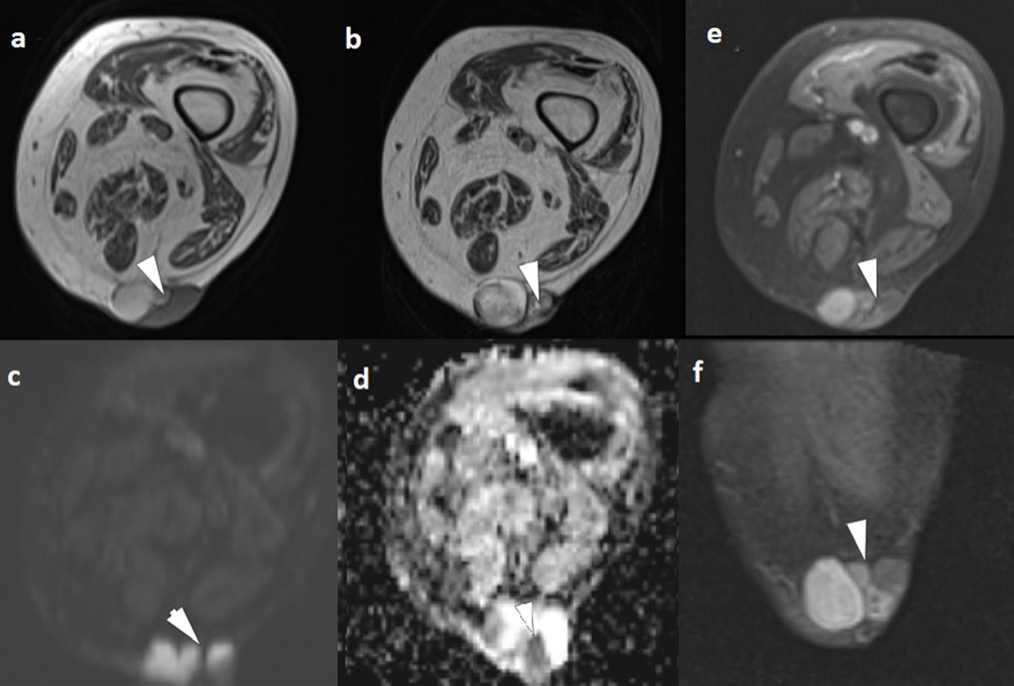
MRI of the mass in axial *T*_1_WI (**a**) and *T*_2_WI (**b**) showing that the solid components of the mass display low signal intensity. On DWI, they show hyperintensity (**c**) with a low ADC value (**d**). After gadolinium-based contrast agent administration, we note mass enhancement on axial and coronal planes (**e, f**). ADC, apparent diffusion coefficient; DWI, diffusion-weighted imaging.

**Figure 4.**
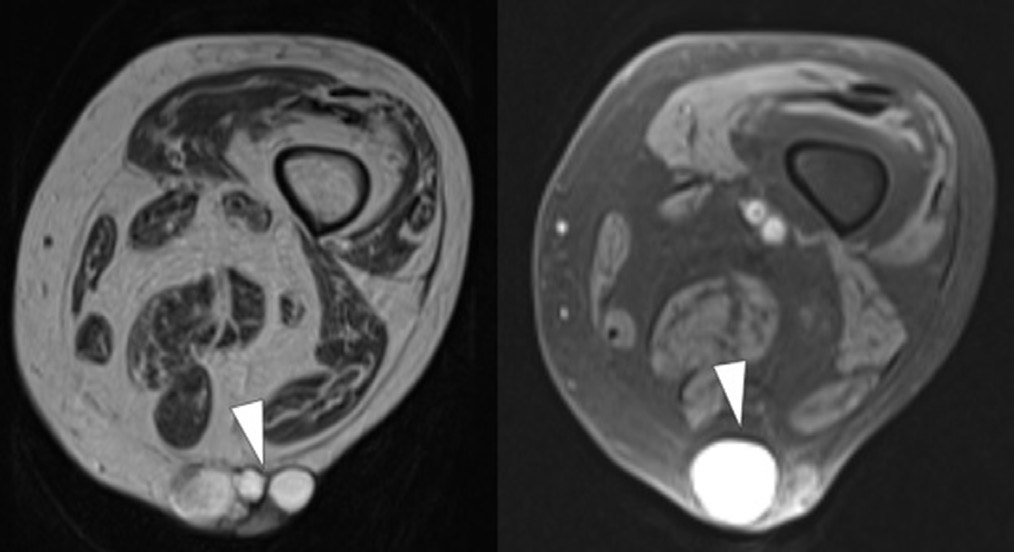
Axial MRI of the skin mass showing the cystic components with heterogeneous signal on *T*_2_ weighted images (**a**) some of these cysts show high signal intensity on *T*_1_ weighted (FS) images (**b**). FS, sat-saturated.

**Figure 5.**
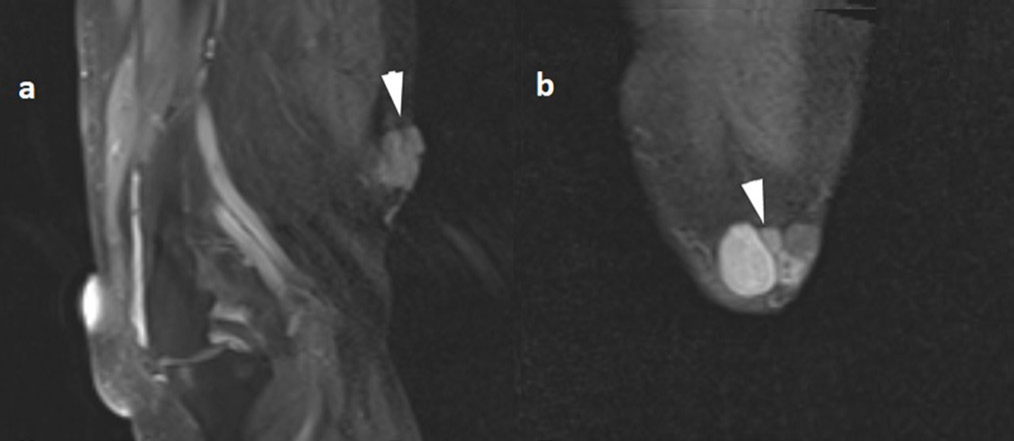
Coronal (FS) MRI (a) and sagittal (FS) MRI of the skin mass after administration of contrast.

An ultrasound was performed. It showed a multilobulated, well-defined, oval-shaped lesion of the dermis and subcutaneous tissue, with solid and cystic components. The solid components were hypoechoic and heterogeneous with increased peripheral vascularity, and the cystic components had echoic spots within them (Figure 2). MRI of the thigh showed a well-marginated, multilobulated lesion, within the cutaneous and subcutaneous fat tissue, with solid and cystic components, measuring 4.6 × 4 × 3 cm.The solid components were hypointense in *T*_1_WI, *T*_2_WI, on DWI they show hyperintensity with a low ADC value and they showed contrast enhancement. The cystic component consisted of multiple cystic lesions. Some of these lesions had heterogeneous signal intensity with high signal intensity on *T*_1_ weighted images, suggestive of hemorrhage or high protein content. No evidence of surrounding invasion was found (Figures 3–5) . A CT scan of the chest, abdomen, and pelvis was performed and no evidence of distant metastases was found. Skin biopsy results were suggestive of malignant adnexal cutaneous porocarcinoma.

### Treatment

**Figure 6.**
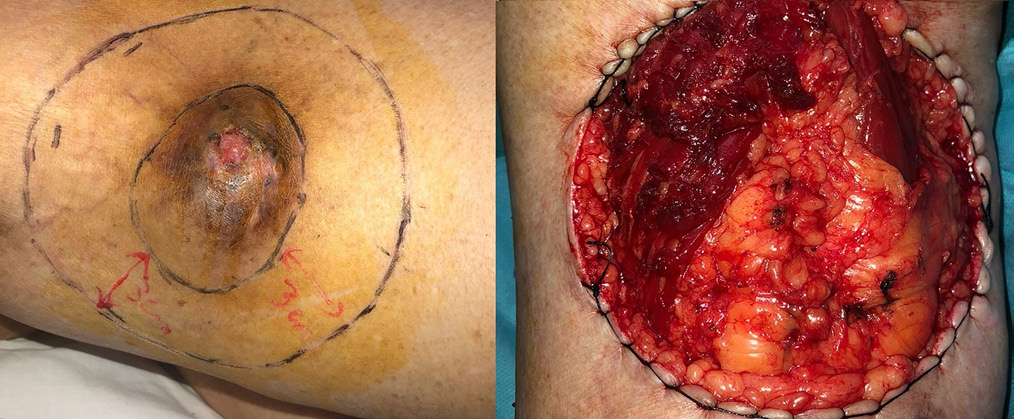
Per-operative photography of the surgical procedure: elliptical incision with wide margins of 3 cm.

**Figure 7.**
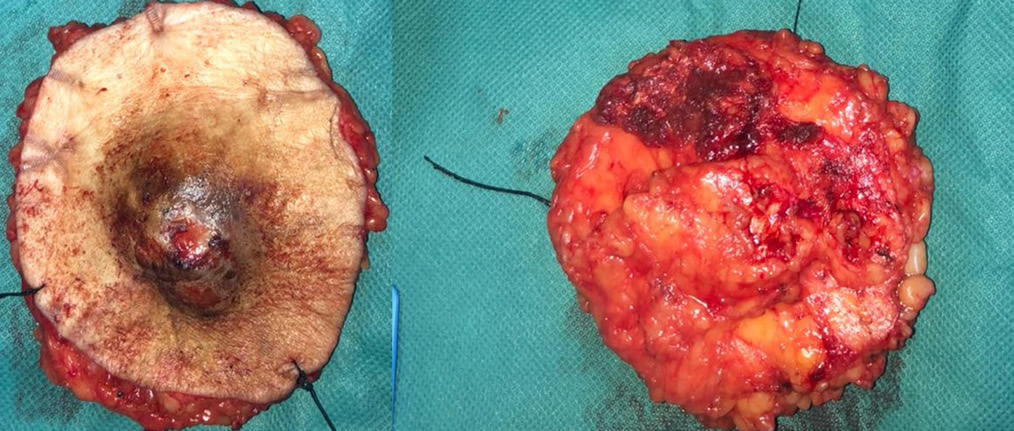
Photography of the excised mass, the outer surface of the mass was brownish tan and nodular.

Given that it’s a localized form, with no evidence of lymphatic or distant metastasis, the multidisciplinary medical committee decided on wide local excision through a transverse elliptical incision. Surgical excision of the tumor with wide margins of 3 cm was performed (Figures 6 and 7). Sentinel lymph node biopsy was negative. Histopathological analysis of the tumor confirmed the diagnosis of eccrine porocarcinoma with high mitotic activity. Histological margins were clear.

### Histopathology

Histopathological examination revealed solid aggregates of epithelial tumor cells of small size replacing the epidermis and growing in bands deep in the adjacent dermis. They also showed nuclear pleomorphism along with a high mitotic activity and abnormal mitotic figures. We also note the presence of abortive ductal differentiation. Some of these cells contained glycogen.

### Outcome and follow-up

The patient was kept afterward until the wound healed completely and no signs of infection or complications were noticed. She was then discharged with planned annual follow-up appointments.

### Discussion

Eccrine porocarcinoma, first described by Pinkus and Mehregan in 1963, is an extremely rare malignant cutaneous tumor that originates from eccrine sweat glands.It represents 0.005% of all epithelial cutaneous tumors.^1^ According to a meta-analysis of 453 cases by Abdulwahid M. Salih et al, there is no age predilection and the mean age of presentation is 67 years. Risk factors for developing this tumor are still not well understood. Masses and nodules are the most frequent clinical presentation.^2^ It may appear as an original tumor or arise from the transformation of a pre-existing benign poroma. Lower extremities are a commonly involved location. However, cases were also reported in the upper extremities, abdomen, face, and scalp. Due to its aggressive character, the recurrence rate is frequent, as well as lymphovascular invasion and distant metastases.^1–3^ The most frequent sites to which porocarcinoma metastasizes are lymph nodes, lungs, liver,and brain. Cases with metastases have a poor prognosis compared to localized forms.^2^ The contribution of imaging in the management of eccrine porocarcinoma is undeniable. The treatment plan depends mainly on the local invasion and metastatic status. Few are the reports in the literature that discuss MRI features of this tumor. A case report published in 2019 by the Japanese Society for Magnetic Resonance in Medicine, described a pedunculated mass of the temporal region diagnosed as eccrine porocarcinoma. On *T*_2_ weighted images, the tumor showed a central lesion with low signal intensity and a peripheral lesion with high signal intensity, as well as small cysts with hemorrhaging in some parts. The enhancement was rapid and persistent.^4^ Cunningham et al reported a small lesion at the heel of the foot that showed slight hyperintensity on T1 and *T_2_* weighted images compared to the rest of the skin, with no signs of extension to the adjacent structures.^5^ A large porocarcinoma of the perineal region was published in Abdominal Imaging, the mass had undefined margins, and it had intermediate to high signal intensity on *T_2_* weighted images with non-homogeneous enhancement after contrast administration. This time the mass was invasive and it infiltrated the anal canal, the distal vagina and vulva, the urethral orifice, and the perineal soft tissue.^6^ Diffusion-weighted images are not very contributive because of susceptibility artifacts. This tumor is usually misdiagnosed or diagnosed at a late stage because the clinical presentation is vague and also there isn’t enough background in the literature about its radiological characteristics. Histological confirmation is mandatory for the diagnosis. Nonetheless, imaging plays an important role. Before a biopsy, an MRI can contribute to defining the malignant nature of the tumor by detecting local extension and lymph nodes involvement. According to cases found in the literature as well as our case, the MRI appearance can be variable. However, we conclude that these tumors have strong and rapid enhancement, and also the presence of cystic components and hemorrhaging is not rare. After biopsy confirmation, imaging helps the surgeon determine the margins and the depth of the excision. Imaging modalities are also fundamental to detect distant metastases. Our case presented a local form of eccrine porocarcinoma, a biopsy was performed before MRI and the surgeon asked whether there was an invasion of the adjacent muscle and aponevrosis. In the absence of the latter, a wide surgical excision was performed given the aggressive nature of the tumor and the high risk of local recurrence.

### Features on MRI for differential diagnosis

The pedunculated configuration is mostly seen in adnexal cutaneous tumors. Cutaneous squamous cell carcinoma, cutaneous malignant melanoma and cutaneous angiosarcoma present as flat elevated lesions. A homogenous T2 signal might be characteristic of porocarcinoma, cutaneous lymphoma and cutaneous basal cell carcinoma as opposed to other malignant skin tumors that usually present a heterogeneous signal on *T_2_* weighted images. Hyperintensity in *T_1_* weighted images is noted in porocarcinoma (as was mentioned in our case) and also in cutaneous malignant melanoma. Both porocarcinoma and cutaneous basal cell carcinoma may include cystic components.^4,7,8^

## Learning points

- Porocarcinoma is a rare and poorly known type of skin neoplasm that can be challenging to diagnose due to the clinical similarity to other skin tumors.
- On MRI signal intensity is variable; however, these tumors have strong and rapid enhancement and also the presence of cystic components and hemorrhaging is not rare.
- MRI can determine the local extension and lymph node involvement, thus guide the surgeon in the excision process.
- Imaging is also crucial to look for distant metastasis which changes the treatment plan.
- An early detection and wide surgical excision can minimize mortality given the aggressive nature of this tumor.
